# Factors Associated with Uptake of Effective and Ineffective Contraceptives among Polish Women during the First Period of the COVID-19 Pandemic

**DOI:** 10.3390/ijerph191912748

**Published:** 2022-10-05

**Authors:** Zbigniew Izdebski, Krzysztof Wąż, Damian Warzecha, Joanna Mazur, Mirosław Wielgoś

**Affiliations:** 1 Department of Biomedical Aspects of Development and Sexology, Faculty of Education, Warsaw University, 00-561 Warsaw, Poland; 2 Department of Humanization in Medicine and Sexology, Collegium Medicum, University of Zielona Gora, 65-729 Zielona Gora, Poland; 3 Institute of Pedagogy, Faculty of Social Sciences, University of Zielona Gora, 65-729 Zielona Gora, Poland; 4 1st Department of Obstetrics and Gynecology, Medical University of Warsaw, 02-015 Warsaw, Poland

**Keywords:** contraception, COVID-19, family planning services, Poland, social determinants

## Abstract

The COVID-19 pandemic has burdened the healthcare system and influenced individuals’ health-related choices. The aim of the study was to estimate the prevalence and to identify the correlates of the use of more and less effective contraceptive methods among Poles in the initial period of the COVID-19 pandemic. The cross-sectional online study was conducted among the representative sample of 642 female respondents aged 18–49. Three groups of contraception choices (only effective methods—42.2%, mixed effective and ineffective methods—26.8%, none—31.0%) were distinguished and 11 potential determinants were considered. One in ten women declared having difficulty in accessing contraception during the first months of the pandemic. A multinomial logistic regression model explained 48.7% of the variation in contraceptive method choice. Both effective and ineffective methods were more often declared by young women, and less often in case of lower education, planning children or subjective no need for contraception. In addition, factors that reduced the chance of effective contraception were poor financial situation, already having children and a relatively higher degree of religiosity. The study confirmed that a significant share of Polish women do not opt for effective methods of contraception. Their choices had strong demographic, social and cultural determinants.

## 1. Introduction

Conscious family planning, including facilitating access to contraception and improving sexual education, constitutes one of the basic assumptions of strategies to improve reproductive health [1].

Epidemiological analyses carried out in the USA show that the percentage of unplanned pregnancies in the general population may be as high as 45% [2]. Moreover, 40% of women who have an unplanned pregnancy decide to terminate it [3]. Worldwide, it is estimated that 68,000 women die annually due to complications from “unsafe” pregnancy terminations [4]. Attention should also be paid to the increased risk of sexually transmitted diseases and cervical cancer in patients who do not use any methods of birth control [5].

Population studies conducted in Poland since 1997 show improvement in the use of contraception methods. We observe an increased use of effective methods and those that additionally protect against sexually transmitted diseases (condoms). However, there is still a high percentage of couples not using any reliable contraception [6].

Choosing the right method of contraception is usually a complex process that requires taking into account an individual’s preferences and procreation plans, health issues and the expected effectiveness of the particular methods [7]. “Long-acting reversible contraceptives” (LARC), such as intrauterine devices (IUD) or subdermal implants, have the lowest failure rates of all methods (1%), while condoms and withdrawal carried the highest probabilities of failure (13% and 20%, respectively) [8]. Compared with the latter methods, long-acting reversible contraceptives do not require any additional measures on the part of the patient to achieve effectiveness. This is one reason these methods are the most effective (99%) method of contraception [9].

An important background for assessing the level of access to contraception in Poland compared to other countries is the periodically (since 2017) published Contraception Policy Atlas Europe, an initiative of the European Parliamentary Forum for Sexual and Reproductive Rights (EPF). The purpose of these studies is to track government policies on access to contraceptive supplies, family planning counselling and the provision of online information on contraception. All countries are analysed on the basis of 16 criteria, sub-divided into the three categories mentioned above, each country is allocated an overall score which corresponds to a specific colour ranging from green to light green for the best scoring countries, to yellow, orange and red for the worst performers. In light of the latest fifth edition of this atlas launched on February 2022, Poland ranks last among the 46 countries in the WHO European Region. Eastern EU countries, including some of Poland’s neighbours (Czech Republic, Lithuania, Slovakia), are mentioned among the worst performing states [10].

The COVID-19 pandemic has caused a global crisis. This situation has worsened access to healthcare, including access to prescription medicines [11,12]. It is worth emphasizing that in Poland at present, hormonal methods of contraception, including emergency contraception, are only available by prescription.

With the onset of the COVID-19 pandemic, most professional health associations and organisations published recommendations on the need for proper contraceptive counselling to be available, emphasizing the necessity of ensuring continued access to effective methods of contraception [13]. Nevertheless, the pandemic could make contraceptive counselling difficult. The pandemic could also be responsible for changing reproductive plans, which particularly affects the poorer social groups [14,15].

The aim of the current study was to assess contraception selection factors and the use of contraceptive methods by women in the initial period of the COVID-19 pandemic.

The study was based on the following research questions:

1. How often do women choose effective and ineffective contraceptive methods?
2. Which demographic, social, cultural, and sexual factors correlate with decisions about the choice of more or less effective family planning method in the univariate analysis?
3. Which variables predict that choice in the multivariate analysis?

## 2. Materials and Methods

### 2.1. Study Population

The data were collected as part of the project “Health and sexual life of Polish adults in the times of COVID”. The online survey was conducted at the end of May and the beginning of June 2020. The project was approved by the Scientific Research Ethics Committee of the Faculty of Education of the University of Warsaw (opinion no. 6/2020).

Our study population was selected from a nationwide panel sample of 100,000 registered by an external contractor (IQS Company). The national representativeness of the sample was structured by sex, age, and place of residence (voivodship, population size of the town). The research survey remained open until the target sample of 3000 respondents was reached. Among them, 892 women aged 18–49 were identified, and then a subgroup of 642 women was selected who described themselves as heterosexual or bisexual, who were not pregnant at the time of the survey and had engaged in sexual activity in the 3 months prior to the survey. Among them, 450 women indicated they had used some form of contraception and marked all the methods that concerned them at the time.

### 2.2. Questionnaire

The research tool was an original questionnaire consisting of 16 thematic sections on taking up sexual activity, contraception, relationship functioning and sociodemographic data.

The survey participants were asked; *What methods of pregnancy prevention have you (you and your partner) used in the past 3 months?* Respondents could choose from a list of ten methods or enter other, unlisted methods. Contraceptive methods were divided into two groups, characterised by high and low effectiveness, according to WHO recommendations [16], and classified using the Pearl Index (defined as the number of unintended pregnancies within one year, in 100 woman who use a particular method) [17].

The demographic and social characteristics surveyed included: age, level of education, size of the town (in 7 categories, coded into two groups), employment status and their household economic status. In assessing their economic situation, respondents answered to what extent they could meet their daily needs, whether they could afford to spend more, or whether they could make savings and investments. They were divided into three groups corresponding to a bad, average or good, and very good material situation. In determining attitudes to faith, respondents’ declarations about religious practice were also considered. In addition, variables related to relationships and relationship status, the number of sexual partners, having children and childbearing plans, as well as any difficulties experienced in accessing contraceptives during the pandemic were considered.

### 2.3. Statistical Analysis

Descriptive statistics are given as frequencies and percentages. The main outcome was the division of the study population into three groups according to the effectiveness of contraception (i.e., high-performing methods, either low-performing methods or both effective and ineffective methods, and not using contraception). The Pearson chi-square test was used to compare these groups in relation to demographic, social and related to sexuality or reproductive health factors. When interpreting results presented in larger contingency tables, we analysed adjusted standardized residuals, where the absolute value > 1.96 corresponds to a significance of *p* < 0.05. In the multivariate analysis, the multinomial logistic regression model was estimated, with no users as the reference category. Goodness-of-fit was estimated by calculating Nagelkerke pseudo *R*^2^, where the value above 0.7 is recommended [18]. Regression analysis results are presented as adjusted odds ratios and 95% confidence intervals (CI).

The statistical software IBM SPSS Statistics for Windows, v. 26.0. (IBM Corp., Armonk, NY, USA) was used for analysis.

## 3. Results

In the group of 642 respondents, the mean age was 33.9 years (SD = 8.8). Most of the respondents declared that they were believers (81.0%, with 35.5% being non-practitioners) and were in a permanent relationship lasting at least 6 months (91.9%). Most of the surveyed women had children (73.7%). Detailed data on the respondents are presented in Table 1.

Table 2 presents the share of not mutually exclusive contraceptive methods used in the last three months, broken down into high and low effectiveness methods. The most frequently used method of contraception in the study group was a condom (63.2%). Among the highly effective methods, hormonal agents were also used relatively often—34.2% (most often in the form of tablets). In the case of low-efficiency methods, the most common was the intermittent intercourse (30.4%). So-called natural methods were indicated by 12.5% of the respondents.

Table 3 contains information on the preferences regarding the contraception in three predefined groups and according to selected factors.

A post hoc analysis using the adjusted residuals method identified factors for which the frequency of effective, ineffective or contraceptive avoidance methods differed significantly from the population average. Positive residuals here means a frequency higher than expected. It was shown that the frequency of use of effective methods was particularly high compared to the surveyed population among women: with higher education (rest 3.6), in good financial situation (2.8), non-believers (3.2) and not planning to have children (2.9). Ineffective methods were particularly common compared to other groups among women with a high school education (rest 3.1), believers but irregular practitioners (2.4) and those planning procreation but not in the near future (2.4). Contraception abandonment was particularly common among: the oldest women aged 42 and over (rest 2.7), believers but not practicing (2.6), planning to have a child in the near future (3.5), and recognizing that they do not need contraception (13.2). It is also worth noting the groups where contraception avoidance was noted to be much less frequent than the population average. In this case, the adjusted residual was high but negative. To be mentioned here are women: the youngest, i.e., under 25 years of age (the rest −3.2), with secondary or higher education (−3.9 and −3.4, respectively), urban residents (−3.1), living in good or very good material conditions (−2.8), studying (−2.7), having one partner in life (−2.9), non-believers (−2.7), planning a pregnancy a little later (−3.1, and reporting no difficulties in accessing contraception (−11.2).

The results of the multinomial logistic regression model estimation can be found in Table 4. They allow to identify factors that increase the chance of using effective (model 1) and ineffective (model) contraceptive methods, taking as a reference group women also sexually active but not using any birth control methods. In both models, the chance increased in the youngest group compared to the oldest one, but the effect was stronger in model 1. In both models, the chance also decreased in the group with less than secondary education, compared to a higher education, in the case of wanting to have a child in the next two years and in the case of declaring no need for contraception. Only in model 1 was it shown that less frequent use of effective methods is associated with living in rural areas, poor financial situation and already having children. There was also an association with attitude toward faith. Compared to non-believing women, believing and non-practicing women were significantly less likely to use effective contraception. According to the Nagelkerke pseudo R-sq coefficient, this model explains 48.7% of the variability in the method of contraception choice.

**Figure 1:**
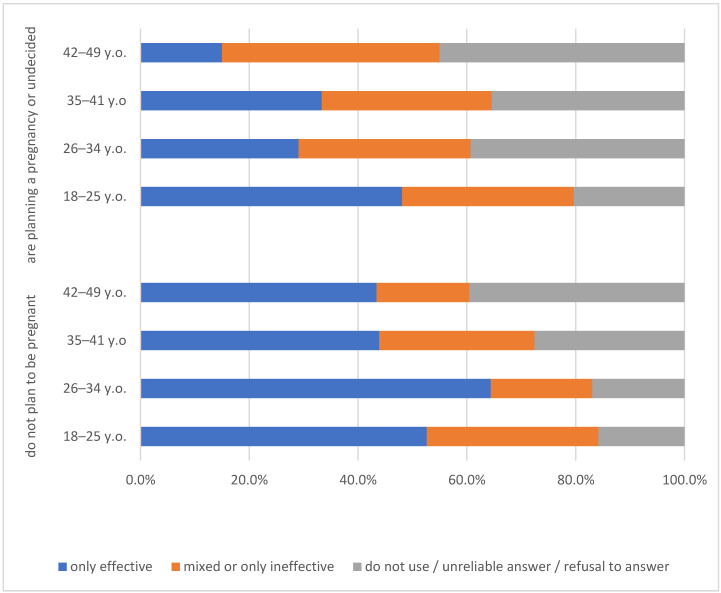
Contraception models in comparison with the age of women not planning and planning to have children.

The contraceptive models (Figure 1) were compared for women not planning and planning to have children with a strong interaction with age confirmed (Figure 1). An effective model of contraception is significantly more often used by respondents aged 26–34 who do not plan to have a child in the next two years and younger ones (64.4% and 52.6%, respectively). At the same time, in the group considering to procreate, the oldest women reported effective methods of contraception less frequently (15.0%) followed by women aged 26–35 (29.1%).

## 4. Discussion

According to the best available knowledge, the presented study is the first in Poland and in this part of Europe that introduced the preferences and predictors regarding the choice of individual contraceptive methods during the COVID-19 pandemic. The advantage of this research is the representativeness of the sample, that allows to generalize the obtained results for the entire population of Polish women of childbearing age.

The most frequently used contraceptive method among the study group was the condom (63.2%), which dominates over the other methods of higher effectiveness, including hormonal agents (34.2%) or the IUD (6% of the respondents). In case of low-efficiency methods, the most frequently used was the intermittent intercourse (30.4%), an extremely unreliable procedure that nowadays should not be considered as a sufficiently effective. Less frequent usage of effective contraceptives seems to be associated with the living in rural areas, poor financial situation and already having children. Moreover, we observed an association with attitude toward faith. Compared to non-believing women, believing and non-practicing women were significantly less likely to use effective contraception. The obtained results suggest that there is still a significant relationship between the question of faith and the usage of particular method of contraception.

Natural disasters and epidemics worsened the availability of and women’s access to healthcare providers and often resulted in lower use of family planning, particularly methods requiring facility-based interaction [19]. Contraceptive counselling during remote consultations does not always seem to be effective [20]. A mathematical model predicted a 10% decline in the use of short and long-acting contraceptive methods in low- and middle-income countries due to reduced access [21]. Despite a disruptive event such as the COVID-19 pandemic, it is essential to ensure that women can have control over their reproductive life and protect the access to contraceptives and family planning services [22].

In the studies carried out on the nationwide representative sample in 2017, people aged 18–49 who had been sexually active in the last 12 months were asked about contraception as in the reported studies [23]. Compared to studies carried out in 2017, in the first period of the pandemic, women significantly less frequently used ineffective methods, i.e., interrupted intercourse (30.4% vs. 17.5%) and a marriage calendar or other natural methods of family planning (12.5% vs. 9.2%), while slightly more effective methods such as condoms (63.2% vs. 67.0%) and hormonal methods, i.e., contraceptive pills (31.5% vs. 35.5%), patches (1.3% vs. 2.5%) and the vaginal ring (1.1% vs. 3.3%).

Our research has confirmed that reproductive health should be studied in the context of many sociodemographic and cultural factors. The authors of the study from Bangladesh obtained similar results on the impact of socioeconomic and demographic factors on the access to family planning. Results showed that factors such as woman’s age, education level, locality, duration of the marriage, ever having been pregnant and the number of children were significantly associated with the usage and the access to family planning during COVID-19 pandemic [24]. Moreover, only 24.42% of the respondents were using oral contraceptive pills compared to 61.7% before the pandemic. Another study reported limited access to prescription and medications during the COVID-19 outbreak [25]. Despite the social-distancing regulations and the COVID-19 pandemic lockdown, reproductive healthcare needs in adolescents and young adults are not diminished [26]. Another study has demonstrated the impacts of COVID-19 on family planning access, multiple sexual partnerships, transactional sex, and the disruption of maternal and child health services [27]. The authors concluded that interventions leading to the immediate availability of and access to all sexual and reproductive health services should be prioritized.

A recently published cohort study of over 9784 women in the UK evaluated the impact of the COVID-19 pandemic on access to contraception and reproductive plans [28]. The authors emphasized over nine times higher difficulty accessing contraception and two times higher (2.1% vs. 1.3%) proportions of unplanned pregnancies during the lockdown. Another study noted an increase in total demand for self-managed abortion during the pandemic (12% compared with the first 9 months) [29].

On the basis of the obtained results, it can be concluded that the chance of using a more desirable model of contraception as compared to the resignation from contraception decreases in the case of older women (over 35 years of age), people who have less than a vocational education, poor financial situation of the family, and those who do not have children. At the same time, it is difficult to interpret the answers of people who declared that they did not need to get access to birth control methods or refused to answer this question. It should also be carefully interpreted that some factors did not turn out to be significant in the adopted model, such as the type of relationship in which they are currently studied, as it may be related to the age of the respondents (a statistically significant correlation was found between these variables).

Findings from another study aimed at the impact of COVID-19 on family planning services showed discontinuation in a family planning preferred method because of social distancing [30]. All married and cohabiting women were continuing to use their contraceptive method. None had an unplanned pregnancy. On the other hand, 50.5% non-cohabiting or single women had discontinued their short-acting reversible contraceptive method while social distancing, for non-method-related reasons; however, 46.5% non-cohabiting or single women had continued their sexual activity, infringing social distancing rules, and 14.9% had had an unplanned pregnancy, for which they had sought a termination.

The relationship between the age of women and the choice of contraception model drew particular attention during the analysis of the research results. It seems that procreation plans may play an important role here. The comparison of the contraceptive models confirmed this assumption. The study conducted in Turkey revealed that about 32.7% of participants planned to become pregnant before the pandemic, but the number significantly decreased to 5.1% throughout the pandemic, and the rate of contraception use by women significantly also decreased during the pandemic [31].

Since there is an important risk of increasing unplanned pregnancies during the COVID-19 pandemic. In this view, we strongly encourage women and healthcare providers to discuss the access to family planning services as a priority service, especially in women who are older, of lower educational level, of poor financial status and within the couples who do not have children yet.

The study is cross-sectional, which makes it impossible to infer cause-and-effect relationships. A significant proportion of the women used multiple methods of contraception. Therefore, it is difficult to assess which method was the dominant one and what was the reason for using several measures (with different effectiveness) in a relatively short period of time. Third, there is no information on the systematic use of methods considered effective. In the case of condoms, the motive for their use may not be so much greater availability in times of limited access to the health care system, but also the desire to prevent STDs.

## 5. Conclusions

These findings suggest a need for increased attention from medical caregivers for their patients with sexual behaviours that lead to the usage of more effective model of contraception, especially in women who are older, of lower educational level, of poor financial status and within couples who do not have children yet. The use of contraception requires constant monitoring, as population indicators are changing. The most burning goals for the future are to implement comprehensive educational programs and direct the attention of gynaecologists to the issues concerning effective and available contraceptive methods. 

## Figures and Tables

**Table 1:** Baseline characteristics of the studied population (N = 642).

Variable	N	%
**Age**		
18–25 years old (y.o.)	155	24.1
26–34 y.o.	182	28.3
35–41 y.o.	151	23.5
42–49 y.o.	154	24.0
**Education level**		
under vocational	66	10.3
vocational	232	36.1
secondary	212	33.0
higher	132	20.6
**Place of residence**		
country	300	46.7
city	342	53.3
**Attitude towards faith**		
believers who practice regularly	106	16.5
believers who practice irregularly	186	29.0
non-practicing believers	228	35.5
unbelievers	78	12.1
not specified or refusal to answer	44	6.9
**Economic status**		
very bad	39	6.1
relative or average	371	57.8
good or very good	215	33.5
refusal to answer	17	2.6
**Occupation status**		
professional works	333	51.8
learns	45	7.0
runs the house	157	24.5
a pension or not working	89	13.9
refusal to answer	18	2.8
**Relationship status**		
formal	324	50.5
informal	266	41.4
out of the relationship	44	6.9
refusal to answer	8	1.2
**The number of sexual partners in a lifetime**		
one	138	23.3
2–3	238	40.1
4 or more	217	36.6
no data	49	
**Children**		
any	473	73.7
no	169	26.3
**Procreation plans**		
yes, soon (up to 2 years)	122	19.0
yes, I want to have kids, but later	108	16.8
no plans to have children	305	47.5
she did not decide	88	13.7
refusal to answer	19	3.0
**Access to contraception within the last 2–3 months**		
had difficulties	64	10.0
had not any difficulties	285	44.4
did not need	284	44.2
refusal to answer	9	1.4

**Table 2:** The usage of contraceptive methods.

Contraceptive Methods		N *	% from the Whole Sample (N = 640)	% among Users (N = 448)
**Ineffective** **(low effectiveness)**	intermittent intercourse	136	21.3	30.4
	calendar method or other natural family planning method	56	8.8	12.5
	globule/gel/ spermicidal foam	8	1.3	1.8
**Effective** **(high effectiveness**)	condom	283	44.2	63.2
	contraceptive pills	141	22.0	31.5
	intrauterine device	27	4.2	6.0
	emergency contraception	7	1.1	1.6
	patches	6	0.9	1.3
	vaginal ring	5	0.8	1.1
	hormonal injections	4	0.6	0.9
	other	1	0.2	0.2

* Two cases of respondents who selected all 10 methods were eliminated—they were considered as unreliable.

**Table 3:** Usage of contraceptive methods according to selected characteristics of the respondents (N = 642).

	Methods Usage *			*p* Value
	Effective Model of Contraception	Ineffective Model of Contraception	Lack of Contraception or No Data	
**In general**	42.2%	26.8%	31.0%	
**Age**				
18–25 years old (y.o.)	48.4%	31.0%	20.6%	Chi.sq = 14.54
26–34 y.o.	40.7%	26.3%	33.0%	d.f. = 6
35–41 y.o.	40.4%	29.1%	30.5%	*p* = 0.024
42–49 y.o.	39.6%	20.8%	39.6%	
**Education level**				
under vocational	36.4%	27.2%	36.4%	Chi.sq = 49.08
vocational	33.6%	20.7%	45.7%	d.f. = 6
secondary	44.8%	34.4%	20.8%	*p* < 0.001
higher	56.1%	25.0%	18.9%	
**Place of residence**				Chi.sq = 9.49
country	38.7%	24.3%	37.0%	d.f. = 1
city	45.3%	29.0%	25.7%	*p* = 0.008
**Attitude towards faith**				
believers who practice regularly	45.3%	25.5%	29.2%	
believers who practice irregularly	36.6%	33.3%	30.1%	Chi.sq = 19.18
non-practicing believers	39.0%	23.7%	37.3%	d.f. = 8
unbelievers	59.0%	23.1%	17.9%	*p* = 0.014
not specified or refusal to answer	45.5%	25.0%	29.5%	
**Economic status**				
very bad	20.5%	30.8%	48.7%	Chi.sq = 16.27
relative or average	40.2%	26.4%	33.4%	d.f. = 6
good or very good	49.8%	26.5%	23.7%	*p* = 0.012
refusal to answer	41.2%	29.4%	29.4%	
**Occupation status**				
professional works	44.7%	26.2%	29.1%	
learns	44.4%	42.3%	13.3%	Chi.sq = 17.72
runs the house	41.4%	26.1%	32.5%	d.f. = 8
a pension or not working	36.0%	24.7%	39.3%	*p* = 0.023
refusal to answer	27.8%	16.6%	55.6%	
**Relationship status**				
formal	42.6%	25.9%	31.5%	Chi.sq = 4.41
informal	39.5%	28.9%	31.6%	d.f. = 6
out of the relationship	52.3%	22.7%	25.0%	*p* = 0.622
refusal to answer	62.5%	12.5%	25.0%	
**The number of sexual partners in a lifetime**				
one	47.8%	31.2%	21.0%	Chi.sq = 17.47
2–3	41.2%	25.2%	33.6%	d.f. = 6
4 or more	41.5%	28.5%	30.0%	*p* = 0.008
no data	34.7%	14.3%	51.0%	
**Chlidren**				Chi.sq = 3.87
any	39.1%	32.5%	28.4%	d.f. = 2
no	43.3%	24.8%	31.9%	*p* = 0.144
**Procreation plans**				
yes, soon (up to 2 years)	26.2%	29.5%	44.3%	
yes i want to have kids but later	45.4%	36.1%	18.5%	Chi.sq = 32.55
no plans to have children	48.2%	22.0%	29.8%	d.f. = 8
she did not decide	40.9%	30.7%	28.4%	*p* < 0.001
refusal to answer	36.8%	15.8%	47.4%	
**Access to contraception within the last 2–3 months**				
had difficulties	60.9%	31.3%	7.8%	Chi.sq = 229.21
had not any difficulties	66.3%	25.6%	8.1%	d.f. = 6
did not need	14.4%	27.5%	58.1%	*p* < 0.001
refusal to answer	22.2%	11.1%	66.7%	

* Ineffective model involves usage of only ineffective methods and combining ineffective methods with effective ones; two cases of respondents who selected all ten methods were eliminated—they were considered as unreliable.

**Table 4:** Estimation results of polynomial logistic regression models.

	Model: Only Effective Model of Contraception				Model: Only Ineffective Model of Contraception			
	*p*	AOR	95% CI(AOR)		*p*	AOR	95% CI(AOR)	
			Lower Limit	Upper Limit			Lower Limit	Upper Limit
**Age**								
18–25 years old (y.o.)	0.000	12.97	4.42	38.07	0.003	4.62	1.68	12.76
26–34 y.o.	0.033	2.48	1.08	5.71	0.096	2.00	0.88	4.54
35–41 y.o.	0.080	1.97	0.92	4.21	0.034	2.22	1.06	4.62
42–49 y.o. (ref.)		1.00				1.00		
**Education level**								
under vocational	0.037	0.33	0.12	0.94	0.257	0.57	0.21	1.51
vocational	0.002	0.29	0.13	0.64	0.028	0.42	0.20	0.91
secondary	0.421	0.72	0.32	1.62	0.844	1.08	0.50	2.36
higher (fer.)		1.00				1.00		
**Place of residence**								
country	0.355	0.77	0.44	1.34	0.092	0.63	0.37	1.08
city (ref.)		1.00				1.00		
**Economic status**								
very bad	0.001	0.11	0.03	0.42	0.098	0.41	0.14	1.18
relative or average	0.052	0.56	0.31	1.01	0.292	0.74	0.42	1.30
good or very good (ref.)		1.00				1.00		
**Children**								
no	0.022	0.41	0.19	0.88	0.543	0.80	0.40	1.63
any (ref.)		1.00				1.00		
**Attitude towards faith**								
believers who practice regularly	0.087	0.40	0.14	1.14	0.455	0.67	0.24	1.89
believers who practice irregularly	0.060	0.40	0.15	1.04	0.914	0.95	0.37	2.42
non-practicing believers	0.017	0.32	0.13	0.82	0.140	0.50	0.20	1.25
unbelievers (ref.)		1.00				1.00		
**Procreation plans**								
yes, soon (up to 2 years)	0.000	0.11	0.05	0.27	0.037	0.44	0.20	0.95
yes, I want to have kids but later	0.134	0.46	0.17	1.27	0.782	1.14	0.44	2.99
did not decide	0.399	0.70	0.30	1.61	0.401	1.40	0.64	3.06
no plans to have children (ref.)		1.00				1.00		
**Access to contraception**								
did not need	0.000	0.03	0.02	0.06	0.000	0.14	0.08	0.26
had difficulties	0.719	1.25	0.37	4.25	0.442	1.63	0.47	5.67
had not any difficulties (ref.)		1.00				1.00		

## Data Availability

The data presented in this study are available on request from corresponding author. The data are not publicly available due to polish law regulation does not allow for public access of medical data.
